# The True Law of Population Based upon Physiology and Psychology

**Published:** 1887-05

**Authors:** Nathan Allen


### The True Law of Population Based upon Physiology and Psychology.

BY NATHAN ALLEN, M.D., LL.D.

In 1798, R. T. Malthus published his great work on the "Principle of Population." He had spent several years in its preparation, and his immediate object was to combat the doctrines of Condorcet and Godwin, who held that the human race was perfectible, and was advancing towards an ideal standard of excellence. Malthus' views were very different. His leading principle was that "population, when unchecked, increases in a geometrical ratio, while subsistance increases only in an arithmetical ratio." He held that "population is necessarily limited by the means of subsistence," and "invariably increases where those means increase, unless prevented by some very powerful and obvious check." He divides these checks into two classes, the positive and the preventive; among the former are wars, famine, diseases of all kinds, unhealthy occupations, extreme poverty, great cities, etc.; and in the latter class are abstinence from marriage and sexual intercourse, as well as from considerations of prudence. The last class come more directly under the control of human agency.

The next writer of any note on population was Thomas Doubleday, who published, in 1840, a work which attempted to prove that there was such a law "connected with the food of the people;" that whenever an individual or species was endangered, a corresponding effort is made by nature for its preservation by an increase of fertility or fecundity, and thus the "deplethoric state" is favorable for increase.

The next writer on this subject is Herbert Spencer, who, in 1852, introduced "a new theory on population." He maintained that an antagonism exists between individualism and reproduction; that matter in its lower forms, for instance, of vegetables, possesses a stronger power of increase than in all higher forms; that the capacity of reproduction in animals is in an inverse ratio to their individualism; that the ability to maintain individual life and that of multiplication vary in the same manner also, and this ability is measured by the development of the nervous system.

While the theories of Malthus and Doubleday are based principally upon objects external to the body, that of Spencer depends more strictly upon physical organization. Fourier and some French writers have advanced the idea that "just in proportion as individuals become advanced in civilization, in the same proportion the race inclines to run out;" but whether this depends upon some change in physiological laws or upon the influence of external agents, we are not informed. In establishing any such law or general principle it is highly important to understand distinctly what this principle is, and what is its basis. Most writers make it depend upon food, climate, government, state of society, epidemics, war, etc., etc.; but these factors operate mainly outside and independent of the body. It would seem more consistent with common sense and all natural phenomena, that the law which governs the existence, growth, and changes of a living being, should have its basis and development in that same organization. From observation and analogy, we believe such a doctrine exists throughout the whole animal and vegetable creation. The truth of this principle is strikingly illustrated in the improvement and changes that have taken place in domestic animals. The human system cannot be made an exception to a universal principle.

This law of increase or propagation—the most important of all laws—must, in the very nature of things, be inherent in the body; must be incorporated into its very existence, though in its operations it may be affected by extraneous causes and influences. However powerful may be the effect of climate, food, and other external agents upon the application or working of this law, whether to impede, thwart, or modify its operation, the law must exist, we believe, in the body itself, and in a great measure control it. The various changes to which the human body is subjected cannot happen by chance or accident; neither can the causes be dissimilar or contradictory in different nations and races; neither can they radically change or vary from one generation to another. Universality and unchangeableness must characterize such a law. The reason why correct principles have not been brought to bear more directly upon the growth and changes of population is that the principles of physiology were not formerly understood. This science, as far as the application of some of its principles are concerned, is in its infancy. One of the most interesting and important of these applications will be found, we believe, in the establishment of a general law of increase.

What, then, is the briefest definition that can be given of this law? *It consists in the perfectionism of structure and harmony of function*; or, in other words, that every organ in the body should be perfect in its structure, and that each should perform its legitimate function in harmony with all others. Though this perfect physical organization is nowhere to be found in nature, we can readily conceive of such a standard, and that there may be all manner of approximations toward it. The nearer this standard is reached, the more completely the law of propagation will be carried out. Such a basis harmonizes with the great fundamental or general laws of Nature, as we find that they are all based upon the highest or most perfect development of her works.

Any other basis or lower standard would reflect upon the Creator of all things, and interfere with the harmony and order which exist in Nature's operations. Thus, in reference to every organ in the human body, there is such a thing as a normal, perfect structure, and, wherever this exists, they constitute a perfect model or standard for the whole system. All diseases interfere at once with the operations of this law, especially those that are considered hereditary. This class of diseases changes with each generation, and sometimes becomes so intensified that they impair the vitality and strength of the system to such an extent as to prevent propagation. There is a class of diseases or weaknesses, described under the head of "sterility," "barrenness," and "impotence," from which strong evidence may be deduced in proof of a general law of increase.

There is a law in physiology favorable to this theory, described by Dr. Carpenter thus: "There is a certain antagonism between the nutritive and reproductive functions, the one being exercised at the expense of the other. The reproductive apparatus derives the materials of its operations through the nutritive system and its functions. If, therefore, it is in a state of excessive activity, it will necessarily draw off from the individual fabric some portion of aliment destined for its maintenance. It may be universally observed that, when the nutritive functions are particularly active in supporting the individual, the reproductive system is undeveloped, and *vice versa.*

Let, therefore, on this principle, any class of organs or any parts of the body be unduly or very much exercised, it requires the more nutrition to support them, thereby withdrawing what should go to other organs. In accordance with this physiological law, if any class of organs become predominant in their development, it conflicts with this great law of increase. In other words, if the organization is carried by successive generations to an extreme, that is, to a high nervous temperament—a predominance of the brain and nervous system—or, on the other hand, to a lymphatic temperament—a predominance of the mere animal nature—it operates unfavorably upon the increase of progeny. Accordingly, in the highest states of refinement, culture, and civilization of a people, the tendency has always been to run out in offspring; while, on the other hand, all tribes and races sunk in the lowest stages of barbarism, and controlled principally by their animal nature, do not abound in offspring, and in the course of time they tend also to run out. The truth of both these statements is confirmed by history. The same general fact has been observed among all the abnormal classes, such as idiots, cretins, the insane, the blind, the deaf and dumb, and to some extent, with extreme or abnormal organizations, such as are excessively corpulent or spare, as well as of unnatural size, either very large or diminutively small.

If this principle is applied to distinct classes in society, some striking illustrations may be obtained. Take the families belonging to the nobility, the aristocracy, or the most select circles, where by inheritance, refinement, and culture, the nervous temperament has become very predominant, it is found that such families do not increase from generation to generation in offspring, and not unfrequently, in time, they become extinct.

A similar result has also followed the intermarriage of relations, from the fact that the same weaknesses or predispositions are intensified by this alliance. On the other hand, in case these relations have healthy, well-balanced organizations,—it may be they are cousins,—they will abound with healthy offspring, and the stock may improve, and not deteriorate, from the mere fact of relationship.

Strong evidences in favor of some general law of propagation are derived from the influences of hereditary descent. The fact that "like begets like," subject to certain variations and conditions, cannot be called in question. The union of two agents possessing similar and dissimilar qualities, constitutes an important condition to which this law of propagation is subject. Now the same evidence that proves the existence of a law of hereditary agency, implies that there is somewhere a general law, of which, each and every part of this agency, is part and parcel; and no one thing will throw so much light upon this whole subject of heredity as the recognition of a general law based upon a proper standard in nature. And without such acknowledgment, all these hereditary agencies are an enigma,—cannot be correctly understood, nor applied in the most successful manner.

Evidences in favor of the theory here advocated are derived from an analogous law in the animal and vegetable kingdoms. It is well known that great improvements have been made within the present century in domestic animals, and this, too, by the application of physiological laws. To such an extent have the results of observation and experiment been here carried, that this process of change and improvement has been reduced almost to a science. The terms here used—"pure blood," "thorough-bred," "pedigree," "breeding in-and-in," and "cross-breeding"—may all be explained by two great leading principles. One is a general law of propagation, based upon a perfect standard, and the other is the law of inheritance, subject to certain conditions. The three first-named terms have originated more from an observance or carrying out the first law—breeding from the best stock; but the two latter terms depend more upon the effects of inheritance. The results of the experiments in producing domestic stock indicate clearly that there must be some settled rules or laws in the process; and, if so, is there not some great general law governing and controlling all others? A similar law of propagation exists in vegetable physiology. It is a fact well attested by gardeners that, in order to produce flowers and fruit, the soil must not be too rich nor too poor; if the plant or tree grows too luxuriously, its branches or roots must be pruned, while, on the other hand, if unthrifty, it must receive better culture and its roots be enriched before it will become fruitful. It is well understood by gardeners that, in order to raise the best fruit and vegetables, the fairest and best-looking seed must be selected. So in setting out plants and trees, the best-looking and well-balanced specimens are always selected. Other facts and illustrations might be cited from this source to prove that some general law governed in the growth and changes of organic life.

Arguments in favor of this law may be drawn from the writings of Charles Darwin. Two of his leading doctrines are "natural selection," and the "law of variability" The former is defined as "an inherent principle in nature amid all its laws and changes, for betterment, for improvement," which may be expressed more correctly in the "normal standard of physiology," and the "law of variability," may be explained by the law of inheritance. Those principles carefully studied and compared throw much light, one upon the other.

The law of population here set forth should certainly interest medical men. It is based upon Physiology, a science which is their special study, and which they should thoroughly understand in all its applications. It is not a mere theory, nor an abstract general principle, but is in itself, *the normal standard* of the body —capable of endless applications. It will enable us to understand far better the nature of man, his duties and responsibilities in relation to himself, to the family, to society, and particularly to his Maker. It presents new views for improvement where there may be found the perfect free agency of man, combined with the highest motives for human improvement. It could not have been intended by the Creator that man should always be ignorant of the great law of propagation—the most important in the universe,—nor that as a moral and accountable being he should be a mere passive agent in the propagation of the species.

It presents a new standpoint in physiology to study female organization as to what is the best physical standard for propagation, and that all the objects and conditions required by this law should be carried out in the best possible manner. There is yet much to learn in this department.

This law of increase should especially interest physicians, as all its operations are so closely identified with their studies, duties, and responsibilities. This same moral standard of physiology constitutes the foundation--the groundwork upon which are based the laws and conditions which result in the highest measures of health and the longest duration of life; or in other words, it constitutes also the Law of Health and the Law of Longevity. No students, no profession, no class of men should be so deeply interested in the character and application of such laws, as medical men, to whose careful and thoughtful consideration, the writer, in conclusion, commends this whole subject.

